# 3-year revision rates of left vs right cephalomedullary nails with a helical blade design

**DOI:** 10.1007/s11845-025-04000-8

**Published:** 2025-07-09

**Authors:** Linda Kelly, Darren Moloney, Denis Collins

**Affiliations:** Trauma and Orthopaedic Department, Beaumont Hospital, Dublin, Ireland

**Keywords:** Cephalomedullary Nailing, Frailty, Hip Fracture, Osteoporosis, Trauma

## Abstract

**Introduction:**

Intramedullary (IM) femoral nailing in the form of cephalomedullary (CM) nailing is a common treatment of intertrochanteric femoral fractures. Previous literature has demonstrated higher revision rates of left intertrochanteric fractures. Helical blade constructs offer a theoretical advantage over a screw design.

**Objectives:**

The objective of this study is to examine 3 year revision rates for right vs left CM nails with helical blade head screw. No current studies exist which compare revision rates in left versus right IM nails over this defined time period.

**Study design and methods:**

Patients who sustained intertrochanteric femoral fracture subsequently treated by CM nailing between July 2021 and December 2022 were retrospectively identified from a database of orthopaedic patients. Patients were stratified based on left-sided or right-sided fracture. Criteria for inclusion were anterograde femoral nails, performed for Intertrochanteric hip fractures. Exclusion criteria included revision nails, retrograde nails, diaphyseal and subtrochanteric fractures, reverse oblique fracture patterns and prophylactic nails. The primary outcome was 3 year revision rate. Relevant frequency statistics were generated for each cohort with odds ratios comparing revision rates between the two groups.

**Results:**

n = 104 patients were included in the final sample, comprising n = 48 left and n = 56 right sided intertrochanteric fractures who underwent CM nailing. n = 4 (3.8%) TFNAs underwent subsequent surgical revision. Incidence of revision was higher in patients undergoing left IM nailing Left: 3/48, 0.063, Right: 1/56, 0.018, Odds Ratio: 3.667, 95% Confidence Interval: 0.37 – 36.47, p = 0.268).

**Conclusion:**

A greater proportion of patients who sustained a left sided intertrochanteric fracture and were surgically treated with CM nailing underwent revision surgery within 3 years of initial surgery compared to right sided CM nails.

## Introduction

Intramedullary (IM) femoral nailing is a common treatment of intertrochanteric femoral fractures. The incidence of hip fractures is rising globally. There has been an 11% increase in the incidence of hip fractures between 2017 and 2021 in at-risk populations [1]. Estimates suggest a further 32% increase in this incidence by 2029 [2]. This highlights the demand and financial burden that hip fractures will have on our future healthcare system.

Cephalomedullary (CM) nailing is a relatively new surgical technique in the treatment of unstable hip fractures, pioneered by Kirschner [3]. CM nails have undergone advancement down through the decades, solid to hollow, fluted to smooth, stainless steel to titanium. Some modern devices have helical blade head screws to achieve fixation proximally. The Trochanteric Fixed Nail Advance (TFNA) by DePuy Synthes is one such example [4].

Previous literature has suggested higher rates of revision for left sided CM nails compared to right sided [5, 6]. This is based on studies investigating revision rates after a maximum of 12 months post-operatively.

The objective of this study is to examine 3 year revision rates for right vs left CM nails with helical blade head screw.

## Methods & materials

Patients who sustained intertrochanteric femoral fracture subsequently treated by CM nailing between July 2021 and November 2022 were retrospectively identified from a database of orthopaedic patients attending Beaumont Hospital. Patients were stratified based on left-sided or right-sided fracture.

Criteria for inclusion were anterograde femoral nails, with helical blade head screw, performed for intertrochanteric hip fractures. Exclusion criteria included revision nails, retrograde nails, diaphyseal and subtrochanteric fractures, reverse oblique fracture patterns and prophylactic nails.

A priori power analysis was conducted to calculate the proposed sample size. Using an α value of 0.05 and proposed power value of 0.80, required n for inclusion was 100 to achieve significance. This was validated based on previously published studies investigating similar samples and outcomes.

The primary outcome was 3 year revision rate. All collected data was imported into SPSS v29.0 [7]. Frequency statistics were generated for each cohort (Left and Right CM nail). Odds ratios comparing revision rates between the two groups were generated. Fischer’s exact tests were conducted to assess for any significant association between sex and revision rates. Threshold for statistical significance was set at p < 0.05.

## Results

N = 104 cases were included in the final analysis, which included n = 48 (46.2%) left sided intertrochanteric NOF fractures and n = 56 (53.8%) right sided intertrochanteric NOF fractures. The patient cohort consisted of n = 77 (74%) females and n = 27 (26%) males. All primary CM nails were conducted by helical blade insertion.

n = 4 (3.8%) CM nails underwent revision. Of these revisions, ¾ (75%) were left sided whilst only ¼ (25%) was right sided. Incidence of revision was higher in left sided CM nails compared to right (Left: 0.063, Right: 0.018, Odds Ratio: 3.667, 95% Confidence Interval: 0.37–36.47, p = 0.268).

30/48 (62.5%) of left sided fractures were female, whilst 47/56 (83.93%) of right sided fractures were female. The breakdown of sex distribution between the two fracture groups was significant (p = 0.015). Of the n = 3 left sided fractures undergoing revision, 2/3 (66.67%) were female whilst 1/3 (33.3%) was male. The patient undergoing right sided revision was female. However, the interaction between sex and incidence of revision was not significant (p = 0.724).

## Discussion

The objective of this study is to examine 3 year revision rates for right vs left CM nails with helical blade head screw. In total, 104 patients were included in the final cohort, of which n = 4 underwent revision. Incidence of revision was 0.038, which is not significantly different to incidence rates as reported in the literature. (0.019) [8]. Revision rates were higher in left sided intertrochanteric NOF fractures repaired by CM nailing compared to right sided CM nails, however, this failed to reach significance (p = 0.268).

Previous literature has supported these findings, with recent articles finding higher rates of left-sided revisions at 1 year [5]. A 2015 article investigating a prospectively maintained database demonstrated increased rotational stability in right-sided trochanteric fractures compared to left-sided [6].

One leading theory proposed in the literature to explain the increased rate of left-sided revisions is the clockwise torque of the lag screw used in most CM nails. Clockwise rotation of the femoral head lag screw on the right side results in more optimal positioning of the femoral neck against the distal fragment. However, on the left side, clockwise screw insertion and the effects of post-operative weightbearing results in upward femoral neck displacement [9, 10]. This causes migration of the femoral neck away from the distal femoral fragment. This contributes to an increased risk of biomechanical instability and need for further revision.

However, no trials currently exist which investigate the rates of revision associated with anticlockwise torque rotation in CM nail insertion. This is an important avenue of interest for future investigation to assess ways to reduce revision rates, especially in left-sided intertrochanteric NOF fractures. Future studies comparing clockwise versus anticlockwise torque rotation in treatment of left and right-sided proximal femur fractures will be crucial in elucidating the impact of torque rotation in post-operative biomechanical stability of these fractures.

Whilst there was an increased prevalence of females in right-sided intertrochanteric NOF fractures, there was no significant association between sex and incidence of revision. There are no studies in the available literature investigating the impact of sex as a significant predictor of future revision of CM nails. A recent study investigating the impact of sex as a covariate on the radiological and clinical outcomes post-nailing found no significant association between sex and post-operative complications, but confirmed a greater risk of post-operative fracture in females [11].

A limitation in this study was that the only demographic factor considered was sex, whilst other potentially confounding factors included age, bone quality, pre-injury functional status, post-surgical mobilisation, etc. were not included. The inclusion of such potential confounding factors could be a direction for future studies to expand on known risk factors for revision. Although the findings of this paper fail to achieve statistical significance, the higher proportion of left compared to right-sided revisions aligns with current literature findings. Although an a-priori power analysis was conducted to determine a suitable sample size to adequately power this study, the fact that only one patient underwent right sided revision limits the ability of these results to attain significance. Further studies investigating greater population sizes in a greater powered setting are required to further validate these results.

TFNA presents the theoretical advantage of promoting greater post-operative stability of extracapsular proximal femur fractures when compared to other potential surgical fixation techniques such as dynamic hip screws (DHS) [5]. Patients undergoing TFNA have demonstrated greater rates of achieving full weight-bearing status in the post-operative period when compared to DHS, which is essential for optimisation of functional outcomes.

Incidence of revision of TFNA is estimated to be 0.019 [8]. Risk factors associated with TFNA failure include pathological fractures, reverse obliquity type fracture patterns and smoking status [8].

## Conclusion

Consistent with current literature, this study found a higher rate of left sided revisions following cephalomedullary (CM) nail insertion for intertrochanteric neck of femur (NOF) fracture. Furthermore, this study found a higher rate of right-sided NOF fractures compared to males, however, no significant difference was found in revision rates between sexes. Understanding the predisposing risk factors for revision may lend to enhanced surgical techniques and optimisation of patient clinical and functional outcomes in such high-risk populations.

## Declarations

We confirm that this work is original and has not been published elsewhere, nor is it currently under consideration for publication elsewhere.
